# Data on elemental concentrations in marine sediments from the South and South West of England

**DOI:** 10.1016/j.dib.2021.106901

**Published:** 2021-02-23

**Authors:** Jonathan Richir, Simon Bray, Tom McAleese, Gordon J. Watson

**Affiliations:** a Institute of Marine Sciences, School of Biological Sciences, University of Portsmouth, Ferry Road, Portsmouth PO4 9LY, United Kingdom; b Chemical Oceanography Unit, FOCUS, University of Liège, Liège, Belgium; c Laboratory of Oceanology, FOCUS, University of Liège, Liège, Belgium; d AQASS Ltd, Hound Road, Southampton SO31 5QA, United Kingdom; e School of Biological Sciences, University of Southampton, Life Sciences Building 85, SO17 1BJ, United Kingdom

**Keywords:** Trace element, Heavy metal(loid), Sediment, Public data repository, Data mining, Benthic, Anti-fouling, Shipping

## Abstract

The present Data In Brief methodological paper details the acquisition, mining and pre-processing of elemental concentration data in marine sediments (coastal and open sea) of Southern England, presented and discussed in the co-submitted Environment International paper entitled: “Three decades of trace element sediment contamination: the mining of governmental databases and the need to address hidden sources for clean and healthy seas” [1]. Elemental sediment concentration data were obtained from the two main UK environmental sources, i.e. the Environment Agency (EA) and the Marine Environment Monitoring and Assessment National (MERMAN) database managed by the British Oceanographic Data Centre (BODC). The merged database is the result of a rigorous data selection-validation process and provides spatially and temporally extensive records of arsenic (As), cadmium (Cd), chromium (Cr), copper (Cu), iron (Fe), mercury (Hg), nickel (Ni), lead (Pb) and zinc (Zn) concentrations for hundreds of sites over 31 years (1983–2013). Additional records of manganese (Mn), aluminium (Al), lithium (Li), tin (Sn) [and tributyltin (TBT)], barium (Ba), antimony (Sb), boron (B), calcium (Ca), molybdenum (Mo), cobalt (Co), selenium (Se), potassium (K), magnesium (Mg), beryllium (Be), vanadium (V), titanium (Ti), sodium (Na), silver (Ag), thallium (Tl) and strontium (Sr) are also included. The full secondary database is hosted in the Mendeley Data repository and the geo-spatial information to map sites is given in supplementary files to the paper. To provide end-users with the relevant context on spatial and temporal coverage, monitoring statistics are given for the nine trace elements (TEs). Site-specific statistics include: the first and last year of sediment monitoring, the number of years monitored, and minimum, maximum, mean and median numbers of years monitored. Also given are summary data on the number of sites monitored each year, from the first records from 1983 to 2013. For the nine TEs (total and strong acid digestion techniques are considered separately for Cr and Fe), monitoring statistics are presented separately for coastal and open sea sites. Data are relevant to diverse end-users to assess the local and regional contaminant loads and to contextualize anthropogenic threats to benthic systems in multiple locations from the French/English Channel, southern North and Celtic Seas.

## Specifications Table

**Table:** 

Subject	Environmental Science – Pollution
Specific subject area	Analysis of elemental concentration data from public repositories to assess the contamination in marine sediments.
Type of data	Figure Table Database
How data were acquired	Data on sediment elemental concentrations were acquired from the Environment Agency and the British Oceanographic Data Centre.
data format	analyzed filtered secondary data
Parameters for data collection	Only data provided by the two UK key public repositories identified were included in the analysis and processing stages.
Description of data collection	Coastal and open sea monitoring data recording sediment sample elemental concentrations were requested from two key UK public repositories: a) the Environment Agency (EA) and b) the Marine Environment Monitoring and Assessment National (MERMAN) database managed by the British Oceanographic Data Centre (BODC). Files were requested for sediment elemental concentration data for all UK marine waters (BODC, .xls(x) extension files); and from the two regions South England and South-West England (EA, .mdb extension files).
Data source location	Principal data sources are hosted by the Environment Agency and the British Oceanographic Data Centre.
Data accessibility	The database is hosted in the Mendeley Data repository (http://dx.doi.org/10.17632/m68k63nnk3.1).
Related research article	Richir J., Bray, S., McAleese T., Watson, G.J., 2021. Three decades of trace element sediment contamination: the mining of governmental databases and the need to address hidden sources for clean and healthy seas. Env. Intern. 149, 106,362. https://doi.org/10.1016/j.envint.2020.106362.

## Value of the Data

• The database provides spatially and temporally extensive records of arsenic (As), cadmium (Cd), chromium (Cr), copper (Cu), iron (Fe), mercury (Hg), nickel (Ni), lead (Pb) and zinc (Zn) concentrations in sediments from coastal and open sea sites for 320 UK sites over 31 years.
• Additional records of manganese (Mn), aluminium (Al), lithium (Li), tin (Sn) [and tributyltin (TBT)], barium (Ba), antimony (Sb), boron (B), calcium (Ca), molybdenum (Mo), cobalt (Co), selenium (Se), potassium (K), magnesium (Mg), beryllium (Be), vanadium (V), titanium (Ti), sodium (Na), silver (Ag), thallium (Tl) and strontium (Sr) represent approximately 13% of the full database (334 UK sites when considering the twenty-nine chemicals).
• The database can be used to assess the contaminant load for specific sites, but also to strengthen and target current and future legislative control measures for anthropogenic contaminant inputs.
• Sediment contamination assessment is necessary to understand potential anthropogenic threats and subsequently for managing contaminant impacts upon benthic habitats and trophic bioaccumulation at local, regional and national levels.
• Information published in this paper is relevant to marine ecotoxicologists, coastal ecologists (practitioners, scientists and policy makers) and government decision makers.

## 1 Data Description

**Fig. 1:**
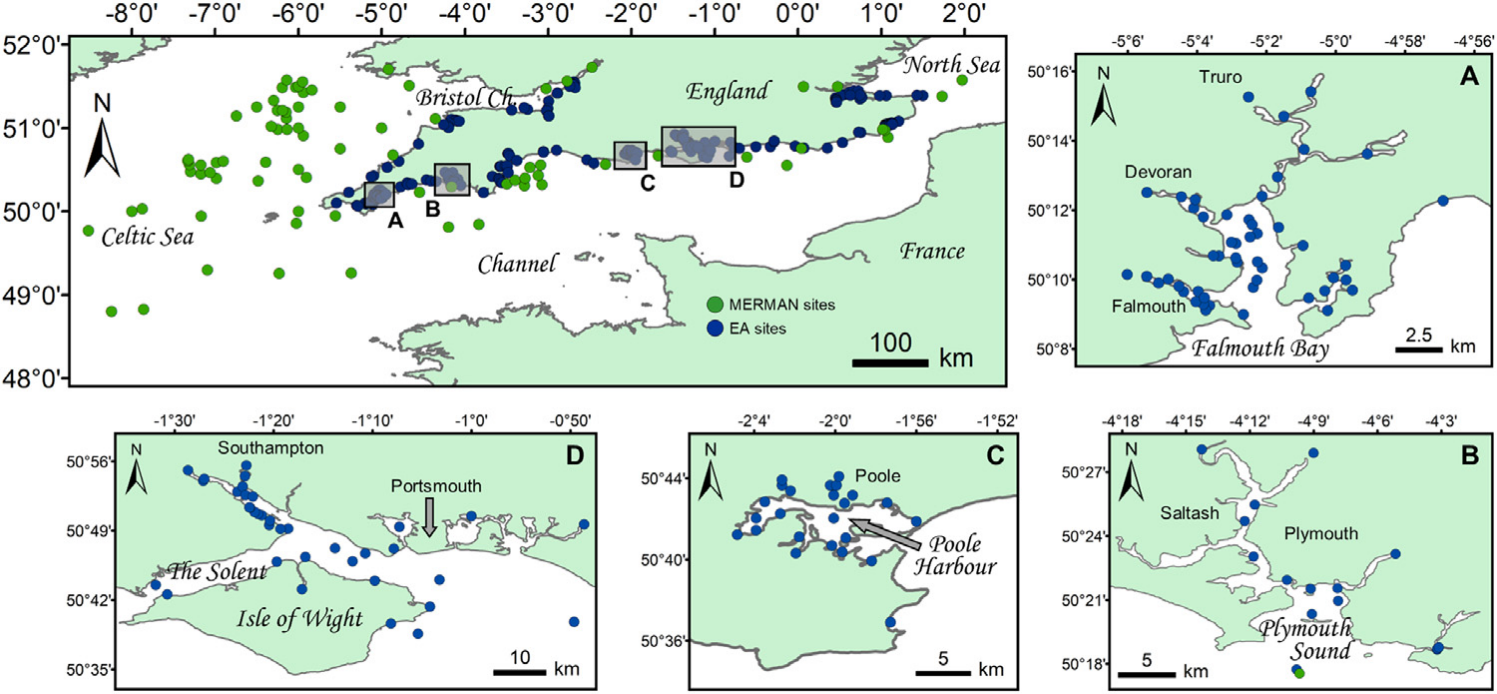
Sites monitored between 1981 and 2013 for elemental concentrations in sediments, with zoom of four large estuary systems of the southern coast of England (UK): A) Falmouth Bay, B) Plymouth Sound, C) Poole Harbour and D) the Solent. Data were requested from the Environment Agency (EA sites, blue circles) and the MERMAN database managed by the British Oceanographic Data Centre (MERMAN sites, green circles). Map created with ArcGIS 10.3.1 (Esri, Redlands, CA), WGS84 projection system.

The secondary data linked to this article and hosted in the Mendeley Data repository (http://dx.doi.org/10.17632/m68k63nnk3.1) provide a summary of >45,000 contaminant concentration data points for twenty-nine marine sediment chemicals from 334 Southern England (UK) sites (Fig. 1, sites within the English/French Channel and the southern North and Celtic Seas), covering a survey period of 31 years (1983–2013). The geo-spatial information contained in geographic data files (.kml extension files, Supplementary Materials) enables end-users to directly visualize and select sites of interest from their geolocalisation on Google Earth (Google LLC). The data were obtained from two UK key public repositories - the Environment Agency (EA), and the Marine Environment Monitoring and Assessment National (MERMAN) database managed by the British Oceanographic Data Centre (BODC) and were subjected to a rigorous selection-validation process. That process is fully described in the Experimental Design, Materials and Methods section of this paper. The secondary data (.csv extension file) have been organized and labelled for interrogation and searching by end-users. Hereafter are given explanations with regard to interpreting the content. The first variable is the Southern England sampling ‘Area’, the second a single number-letter code for each site (‘SITEnb_db’), then ‘Latitude’ and ‘Longitude’ coordinates (WGS84). These are followed by the sampling ‘Location’: either a coastal site (i.e. in transitional, estuarine and coastline waters) or an open sea site (distant/remote from the coastline). With regard to the ‘SITEnb_db’ variable, it has no specific significance except for the _EA or _ME component, indicating the original source, i.e. EA or MERMAN database. The ‘Site Name’ variable is the full site name from the original database (this could be useful for local studies). After the sampling ‘Date’ comes the ‘Dete. Desc.’, the determinand descriptor variable from the original database (‘ME’ character string added for MERMAN determinand descriptors). This variable enables end-users to select data according to the chemical and the related sample processing technique, e.g. grain size fraction used (e.g., <2000 µm or <63 µm) or digestion method (e.g. total hydrofluoric acid specified for EA data). From the categorized data, summary statistics are presented in Tables 1 and 2. The last variables are the ‘Chemical’ name, the concentration or value of the analytical detection limit (DL) when lower [‘Result (ppm, <DL)’], and the concentration with the values under the DL replaced by half of it [2] [‘Result (ppm)’].

**Table 1:** A, B) Number of years (Y) of monitoring effort of trace element contamination in very fine sediments of surveyed sites and C) number of sites monitored for each trace element for six years or more.

Area	Latitude	Longitude	Y.first	Y.last	As	Cd	Cr (sad)	Cr (td)	Cu	Fe (sad)	Fe (td)	Hg	Ni	Pb	Zn	min.Y	max.Y	mean.Y	median.Y
A. number of monitoring years in coastal sites, ordered according to the median, then the mean, then the maximum of monitored years. Statistics are calculated considering strong acid digestion (sad) and total digestion (td) techniques together for Cr and Fe.																			

Channel	50.8454	−0.2869	1990	2007	17	17	17	0	17	0	0	17	17	17	17	0	17	15	17
North Sea	51.3859	1.0569	1996	2013	10	17	16	0	16	16	0	17	16	16	16	10	17	16	16
Plymouth	50.4245	−4.1961	1993	2011	15	15	10	5	15	9	5	15	15	15	15	14	15	15	15
Solent	50.7784	−1.1788	1995	2011	8	17	15	0	15	15	0	17	15	15	15	8	17	15	15
Medway Estuary	51.3081	0.4611	1996	2010	10	15	15	0	15	14	0	15	15	15	15	10	15	14	15
Solent	50.8559	−1.3738	1995	2011	10	17	14	0	14	14	0	17	14	14	14	10	17	14	14
Solent	50.8338	−1.3392	1995	2011	9	17	14	0	14	14	0	17	14	14	14	9	17	14	14
Solent	50.8427	−1.3530	1995	2011	9	17	14	0	14	14	0	17	14	14	14	9	17	14	14
Solent	50.8451	−1.3593	1995	2011	9	17	14	0	14	14	0	17	14	14	14	9	17	14	14
Medway Estuary	51.4294	0.6235	1995	2011	10	17	14	0	14	13	0	17	14	14	14	10	17	14	14
Solent	50.8197	−1.3217	1995	2011	8	17	14	0	14	14	0	17	14	14	14	8	17	14	14
Solent	50.9041	−1.4499	1995	2011	7	17	14	0	14	14	0	17	14	14	14	7	17	14	14
Solent	50.8412	−0.9999	1995	2011	8	17	14	0	14	13	0	17	14	14	14	8	17	14	14
Solent	50.7181	−1.2852	1995	2011	7	17	14	0	14	14	0	17	14	14	14	7	17	14	14
Solent	50.8554	−1.3727	1995	2011	9	17	14	0	14	14	0	14	14	14	14	9	17	14	14
Solent	50.8272	−0.8102	1995	2011	9	17	14	0	14	14	0	14	14	14	14	9	17	14	14
Solent	50.7343	−1.0538	1995	2011	8	16	14	0	14	14	0	16	14	14	14	8	16	14	14
Solent	50.9266	−1.3791	1995	2011	8	17	14	0	14	14	0	14	14	14	14	8	17	14	14
Channel	50.7821	−0.0353	1995	2011	7	17	14	0	14	14	0	14	14	14	14	7	17	14	14
Channel	50.7733	−0.5089	1995	2008	7	14	14	0	14	14	0	14	14	14	14	7	14	13	14
Medway Estuary	51.3649	0.7639	1995	2008	8	14	14	0	14	13	0	14	14	14	14	8	14	13	14
Medway Estuary	51.3482	0.7430	1996	2011	9	16	13	0	13	13	0	16	13	13	13	9	16	13	13
Medway Estuary	51.3952	0.7458	1996	2011	9	16	13	0	13	13	0	16	13	13	13	9	16	13	13
Medway Estuary	51.3683	0.7632	1995	2011	7	17	13	0	13	12	0	17	13	13	13	7	17	13	13
Medway Estuary	51.3295	0.4584	1996	2011	8	16	13	0	13	12	0	16	13	13	13	8	16	13	13
Channel	50.8223	0.5320	1996	2011	7	16	13	0	13	13	0	16	13	13	13	7	16	13	13
Poole Harbour	50.6845	−1.9915	1999	2011	13	13	8	5	13	8	5	13	13	13	13	13	13	13	13
Plymouth	50.3493	−4.1308	1999	2011	13	13	8	5	13	8	5	13	13	13	13	13	13	13	13
Celtic Sea	51.7018	−4.9192	1999	2011	13	12	0	13	13	0	13	13	13	13	13	12	13	13	13
Medway Estuary	51.3104	0.4597	1996	2010	8	15	13	0	13	12	0	15	13	13	13	8	15	13	13
Medway Estuary	51.4464	0.7250	1997	2011	9	15	12	0	12	12	0	15	12	12	12	9	15	12	12
Medway Estuary	51.3884	0.5209	1999	2012	12	12	9	3	12	9	3	12	12	12	12	12	12	12	12
Plymouth	50.3840	−4.1971	1993	2008	12	12	10	2	12	9	2	12	12	12	12	11	12	12	12
Solent	50.9186	−1.4774	1995	2005	5	11	11	0	11	11	0	11	11	11	11	5	11	10	11
Poole Harbour	50.6854	−2.0297	1999	2008	10	10	8	2	10	8	2	10	10	10	10	10	10	10	10
Medway Estuary	51.3345	0.4559	1999	2008	10	10	9	1	10	9	1	10	10	10	10	10	10	10	10
Bristol Channel	51.4708	−3.0245	1999	2008	10	9	0	10	10	0	10	10	10	10	10	9	10	10	10
Bristol Channel	51.5608	−2.7713	1999	2008	10	9	0	10	10	0	10	10	10	10	10	9	10	10	10
Solent	50.7726	−1.2797	1996	2005	4	10	10	0	10	10	0	10	10	10	10	4	10	9	10
Channel	50.7546	−0.7078	1995	2004	3	10	10	0	10	10	0	10	10	10	10	3	10	9	10
Solent	50.6434	−1.0900	2001	2011	9	11	9	0	9	9	0	11	9	9	9	9	11	9	9
Thames Estuary	51.4972	0.0638	1999	2012	9	10	0	9	9	0	9	10	9	9	9	9	10	9	9
Solent	50.8764	−1.3803	1999	2008	9	9	8	1	9	8	1	9	9	9	9	9	9	9	9
Medway Estuary	51.3916	0.6287	2001	2011	8	11	8	0	8	8	0	11	8	8	8	8	11	9	8
Channel	50.9446	0.7502	2004	2011	8	8	8	0	8	8	0	8	8	8	8	8	8	8	8
Bristol Channel	51.7279	−2.4748	1999	2007	8	7	0	8	8	0	8	8	8	8	8	7	8	8	8
Channel	50.7588	0.3515	1996	2009	1	8	8	0	8	8	0	9	8	8	8	1	9	7	8
Celtic Sea	50.2275	−5.3930	1983	2011	9	8	7	0	9	5	0	7	7	6	9	5	9	7	7
Thames Estuary	51.4948	0.4735	1999	2008	7	8	0	7	7	0	7	8	7	7	7	7	8	7	7
Solent	50.7864	−1.1306	1997	2003	3	7	7	0	7	7	0	7	7	7	7	3	7	7	7
Hythe	51.0416	1.0906	1991	2008	5	7	7	0	7	5	0	5	7	7	7	5	7	6	7
Channel	50.9716	1.0431	2005	2010	6	6	0	6	6	0	6	6	6	6	6	6	6	6	6
Dart-Exe-Axe River Estuaries	50.6080	−3.3764	1988	2011	5	6	6	0	6	5	0	5	6	6	6	5	6	6	6
Solent	50.7871	−1.2296	1992	1999	6	6	6	0	6	1	0	6	6	6	6	1	6	5	6
Solent	50.9007	−1.4517	1996	2001	0	6	6	0	6	6	0	6	6	6	6	0	6	5	6
Channel	51.0820	1.2068	1996	2001	0	6	6	0	6	6	0	6	6	6	6	0	6	5	6
Plymouth	50.4118	−4.2040	1987	2012	5	6	6	0	6	3	0	4	6	6	6	3	6	5	6
Bristol Channel	51.2347	−3.0032	1992	2009	4	12	5	0	5	2	0	14	5	5	5	2	14	6	5
Channel	50.7566	0.0679	2004	2011	5	8	5	0	5	5	0	8	5	5	5	5	8	6	5
Solent	50.7255	−1.5317	2004	2010	5	5	5	0	5	5	0	7	5	5	5	5	7	5	5
Bristol Channel	51.1445	−2.9977	1992	2004	4	7	5	0	5	2	0	5	5	5	5	2	7	5	5
Plymouth	50.3656	−4.1709	1983	2008	4	5	5	0	5	2	0	5	5	5	5	2	5	5	5
Bideford Bay Estuary	51.0507	−4.1842	1989	1997	4	5	5	0	5	3	0	4	5	5	5	3	5	5	5
Channel	50.2944	−4.7728	1998	2011	5	5	5	0	5	3	0	4	4	5	5	3	5	5	5
Solent	50.8201	−1.3085	1992	1996	5	5	5	0	5	0	0	5	5	5	5	0	5	4	5
Solent	50.8823	−1.3940	1992	1996	5	5	5	0	5	0	0	5	5	5	5	0	5	4	5
Channel	50.6633	−0.8270	1992	1996	5	5	5	0	5	0	0	5	5	5	5	0	5	4	5
Dart-Exe-Axe River Estuaries	50.5463	−3.4932	1983	2001	5	5	5	0	5	2	0	3	5	5	5	2	5	4	5
Bristol Channel	51.3230	−2.9978	1993	2011	4	11	4	0	4	1	0	9	4	4	4	1	11	5	4
Celtic Sea	50.4131	−5.1261	1995	2010	4	4	5	0	4	2	0	4	5	5	5	2	5	4	4
Poole Harbour	50.7321	−2.0439	1992	2003	3	4	4	0	4	3	0	4	4	4	4	3	4	4	4
Channel	50.3636	−4.4098	1983	2009	4	4	4	0	4	1	0	4	3	4	4	1	4	4	4
Fal Estuary	50.1499	−5.0445	1990	1994	2	4	4	0	4	3	0	2	4	4	4	2	4	3	4
Dart-Exe-Axe River Estuaries	50.7102	−3.0575	1989	1994	1	4	4	0	4	1	0	1	4	4	4	1	4	3	4
Dart-Exe-Axe River Estuaries	50.6904	−3.4873	1990	2011	2	13	2	0	3	1	0	13	3	3	3	1	13	5	3
Bideford Bay Estuary	51.0891	−4.1025	1992	2010	3	8	2	0	3	2	0	14	3	3	3	2	14	5	3
Fal Estuary	50.1917	−5.0275	1995	2011	4	4	2	0	3	4	0	3	4	3	3	2	4	3	3
Channel	50.9833	1.0167	2001	2004	3	3	0	3	3	0	3	3	3	3	4	3	4	3	3
Bristol Channel	51.2052	−3.0205	1994	2008	3	3	3	0	3	0	0	6	3	3	3	0	6	3	3
Channel	50.3190	−4.6815	1994	2013	3	3	3	0	3	3	0	3	3	3	3	3	3	3	3
Channel	50.0719	−5.2981	1984	2006	3	5	3	0	3	1	0	2	3	3	3	1	5	3	3
Bristol Channel	51.1430	−2.9921	1994	2010	3	4	3	0	3	0	0	4	3	3	3	0	4	3	3
Celtic Sea	50.8075	−4.5575	1997	2010	3	3	3	0	3	2	0	2	3	3	3	2	3	3	3
Plymouth	50.4678	−4.2376	1993	1996	3	3	3	0	3	1	0	3	3	3	3	1	3	3	3
Bristol Channel	51.4152	−2.8877	1994	1996	3	3	3	0	3	0	0	3	3	3	3	0	3	3	3
Plymouth	50.3592	−4.1312	2008	2012	3	3	3	0	3	0	0	3	3	3	3	0	3	3	3
Bideford Bay Estuary	51.0385	−4.2564	1990	2010	2	3	3	0	3	1	0	2	3	3	3	1	3	3	3
Dart-Exe-Axe River Estuaries	50.5385	−3.5727	1990	1992	0	3	2	0	3	1	0	1	3	3	3	0	3	2	3
Plymouth	50.3860	−4.0858	1991	2011	1	11	2	0	2	1	0	1	2	2	2	1	11	3	2
Bristol Channel	51.5365	−2.6830	1995	1998	2	2	2	0	2	1	0	3	2	2	2	1	3	2	2
Medway Estuary	51.3984	0.5560	2004	2005	2	2	2	0	2	2	0	2	2	2	2	2	2	2	2
Solent	50.8743	−1.3682	2011	2013	2	2	0	2	2	0	2	2	2	2	2	2	2	2	2
Plymouth	50.2925	−4.1610	2010	2012	2	2	0	2	2	0	2	2	2	2	2	2	2	2	2
Bristol Channel	51.2421	−3.2907	2011	2013	2	2	0	2	2	0	2	2	2	2	2	2	2	2	2
Celtic Sea	50.5987	−4.7963	2005	2008	2	2	2	0	2	2	0	2	2	2	2	2	2	2	2
Channel	50.3411	−4.6954	1997	2006	2	2	2	0	2	2	0	2	2	2	2	2	2	2	2
Channel	50.0993	−5.5433	1993	1994	2	2	2	0	2	1	0	2	2	2	2	1	2	2	2
Bristol Channel	51.4995	−2.7419	1993	1994	2	2	2	0	2	1	0	2	2	2	2	1	2	2	2
Bristol Channel	51.4157	−2.8915	1993	1994	2	2	2	0	2	1	0	2	2	2	2	1	2	2	2
Poole Harbour	50.6719	−2.0325	2004	2005	2	2	2	0	2	0	0	2	2	2	2	0	2	2	2
Poole Harbour	50.7132	−1.9575	2004	2005	2	2	2	0	2	0	0	2	2	2	2	0	2	2	2
Poole Harbour	50.6979	−1.9334	2004	2005	2	2	2	0	2	0	0	2	2	2	2	0	2	2	2
Medway Estuary	51.3595	0.4482	1997	1998	0	2	2	0	2	2	0	2	2	2	2	0	2	2	2
Solent	50.8260	−1.3405	1996	1997	0	2	2	0	2	2	0	2	2	2	2	0	2	2	2
Medway Estuary	51.3390	0.4550	1997	1998	0	2	2	0	2	2	0	2	2	2	2	0	2	2	2
Medway Estuary	51.4468	0.6712	1997	1998	0	2	2	0	2	2	0	2	2	2	2	0	2	2	2
Plymouth	50.3588	−4.1523	1992	1994	1	2	2	0	2	2	0	1	2	2	2	1	2	2	2
Poole Harbour	50.7276	−1.9991	1991	1997	2	1	2	0	2	2	0	1	2	1	2	1	2	2	2
Dart-Exe-Axe River Estuaries	50.3387	−3.5485	1987	1997	1	2	2	0	2	1	0	1	2	2	2	1	2	2	2
Fal Estuary	50.1781	−5.0589	1992	1995	2	1	0	0	2	2	0	0	2	2	2	0	2	1	2
Fal Estuary	50.1846	−5.0502	1992	1995	2	1	0	0	2	2	0	0	2	2	2	0	2	1	2
Fal Estuary	50.2010	−5.0683	1992	1995	2	1	0	0	2	2	0	0	2	2	2	0	2	1	2
Fal Estuary	50.2085	−5.0908	1992	1995	2	1	0	0	2	2	0	0	2	2	2	0	2	1	2
Fal Estuary	50.2064	−5.0743	1992	1995	2	1	0	0	2	2	0	0	2	2	2	0	2	1	2
Fal Estuary	50.1977	−5.0524	1992	1995	2	1	0	0	2	2	0	0	2	2	2	0	2	1	2
Fal Estuary	50.1930	−5.0402	1992	1995	2	1	0	0	2	2	0	0	2	2	2	0	2	1	2
Hythe	51.0402	1.0461	1991	1993	0	2	2	0	2	0	0	0	2	2	2	0	2	1	2
Hythe	51.0434	1.0588	1991	1993	0	2	2	0	2	0	0	0	2	2	2	0	2	1	2
Hythe	51.0463	1.0718	1991	1993	0	2	2	0	2	0	0	0	2	2	2	0	2	1	2
Hythe	51.0529	1.0988	1991	1993	0	2	2	0	2	0	0	0	2	2	2	0	2	1	2
Hythe	51.0559	1.1120	1991	1993	0	2	2	0	2	0	0	0	2	2	2	0	2	1	2
Hythe	51.0593	1.1253	1991	1993	0	2	2	0	2	0	0	0	2	2	2	0	2	1	2
Hythe	51.0316	1.0505	1991	1993	0	2	2	0	2	0	0	0	2	2	2	0	2	1	2
Hythe	51.0356	1.0633	1991	1993	0	2	2	0	2	0	0	0	2	2	2	0	2	1	2
Hythe	51.0382	1.0768	1991	1993	0	2	2	0	2	0	0	0	2	2	2	0	2	1	2
Hythe	51.0446	1.1036	1991	1993	0	2	2	0	2	0	0	0	2	2	2	0	2	1	2
Hythe	51.0476	1.1170	1991	1993	0	2	2	0	2	0	0	0	2	2	2	0	2	1	2
Hythe	51.0507	1.1303	1991	1993	0	2	2	0	2	0	0	0	2	2	2	0	2	1	2
Hythe	51.0236	1.0561	1991	1993	0	2	2	0	2	0	0	0	2	2	2	0	2	1	2
Hythe	51.0266	1.0690	1991	1993	0	2	2	0	2	0	0	0	2	2	2	0	2	1	2
Hythe	51.0296	1.0840	1991	1993	0	2	2	0	2	0	0	0	2	2	2	0	2	1	2
Hythe	51.0326	1.0953	1991	1993	0	2	2	0	2	0	0	0	2	2	2	0	2	1	2
Hythe	51.0359	1.1088	1991	1993	0	2	2	0	2	0	0	0	2	2	2	0	2	1	2
Hythe	51.0393	1.1216	1991	1993	0	2	2	0	2	0	0	0	2	2	2	0	2	1	2
Hythe	51.0424	1.1347	1991	1993	0	2	2	0	2	0	0	0	2	2	2	0	2	1	2
Dart-Exe-Axe River Estuaries	50.5389	−3.5790	1988	1989	0	1	2	0	2	0	0	0	2	2	2	0	2	1	2
Dart-Exe-Axe River Estuaries	50.5409	−3.5636	1988	1989	0	1	2	0	2	0	0	0	2	2	2	0	2	1	2
Bristol Channel	51.5160	−2.7040	1991	2011	2	11	1	0	1	1	0	14	1	1	1	1	14	4	1
Bristol Channel	51.5233	−2.6948	1993	2009	1	8	1	0	1	1	0	12	1	1	1	1	12	3	1
Poole Harbour	50.7128	−1.9925	1993	2004	1	6	1	0	1	1	0	8	1	1	1	1	8	2	1
Channel	50.3769	−4.4640	1999	2010	1	2	1	0	2	2	0	1	1	1	2	1	2	1	1
Bristol Channel	51.5537	−2.6923	1991	1997	2	1	1	0	1	1	0	2	1	1	1	1	2	1	1
Bristol Channel	51.5249	−2.7026	1991	1993	2	1	1	0	1	1	0	1	1	1	1	1	2	1	1
Bristol Channel	51.5190	−2.7076	1991	1993	2	1	1	0	1	1	0	1	1	1	1	1	2	1	1
Fal Estuary	50.1543	−5.0607	2006	2006	1	1	1	0	1	1	0	1	1	1	1	1	1	1	1
Poole Harbour	50.7043	−2.0452	2011	2011	1	1	0	1	1	0	1	1	1	1	1	1	1	1	1
Fal Estuary	50.1520	−5.0625	2006	2006	1	1	1	0	1	1	0	1	1	1	1	1	1	1	1
Fal Estuary	50.1561	−5.0672	2006	2006	1	1	1	0	1	1	0	1	1	1	1	1	1	1	1
Fal Estuary	50.1609	−5.0732	2006	2006	1	1	1	0	1	1	0	1	1	1	1	1	1	1	1
Solent	50.8232	−1.1212	2011	2011	1	1	0	1	1	0	1	1	1	1	1	1	1	1	1
Solent	50.7653	−1.2002	2011	2011	1	1	0	1	1	0	1	1	1	1	1	1	1	1	1
Channel	50.7661	−0.7677	2011	2011	1	1	0	1	1	0	1	1	1	1	1	1	1	1	1
Bristol Channel	51.2239	−3.2499	2012	2012	1	1	0	1	1	0	1	1	1	1	1	1	1	1	1
Fal Estuary	50.1650	−5.0852	2006	2006	1	1	1	0	1	1	0	1	1	1	1	1	1	1	1
Channel	50.8373	−0.2865	2013	2013	1	1	0	1	1	0	1	1	1	1	1	1	1	1	1
Fal Estuary	50.1691	−5.1003	2006	2006	1	1	1	0	1	1	0	1	1	1	1	1	1	1	1
Bristol Channel	51.2133	−3.4402	1993	1993	1	1	1	0	1	1	0	1	1	1	1	1	1	1	1
Fal Estuary	50.1682	−5.0909	2006	2006	1	1	1	0	1	1	0	1	1	1	1	1	1	1	1
Channel	50.3290	−3.4960	2009	2009	1	1	0	1	1	0	1	1	1	1	1	1	1	1	1
Fal Estuary	50.1670	−5.0806	2006	2006	1	1	1	0	1	1	0	1	1	1	1	1	1	1	1
Medway Estuary	51.4215	0.6513	2012	2012	1	1	0	1	1	0	1	1	1	1	1	1	1	1	1
Fal Estuary	50.1580	−5.0632	2006	2006	1	1	1	0	1	1	0	1	1	1	1	1	1	1	1
Fal Estuary	50.1665	−5.0379	2006	2006	1	1	1	0	1	1	0	1	1	1	1	1	1	1	1
Fal Estuary	50.1722	−5.0354	2006	2006	1	1	1	0	1	1	0	1	1	1	1	1	1	1	1
Fal Estuary	50.1771	−5.0481	2013	2013	1	1	0	1	1	0	1	1	1	1	1	1	1	1	1
Fal Estuary	50.1781	−5.0562	2006	2006	1	1	1	0	1	1	0	1	1	1	1	1	1	1	1
Bideford Bay Estuary	51.0031	−4.1997	2013	2013	1	1	0	1	1	0	1	1	1	1	1	1	1	1	1
Fal Estuary	50.1967	−5.0637	2006	2006	1	1	1	0	1	1	0	1	1	1	1	1	1	1	1
Celtic Sea	50.5260	−4.9332	2012	2012	1	1	0	1	1	0	1	1	1	1	1	1	1	1	1
Fal Estuary	50.2051	−5.0676	2006	2006	1	1	1	0	1	1	0	1	1	1	1	1	1	1	1
Helford River Estuary	50.0845	−5.1088	2005	2005	1	1	1	0	1	1	0	1	1	1	1	1	1	1	1
Fal Estuary	50.2160	−5.0281	2006	2006	1	1	1	0	1	1	0	1	1	1	1	1	1	1	1
Fal Estuary	50.2543	−5.0420	2005	2005	1	1	1	0	1	1	0	1	1	1	1	1	1	1	1
Fal Estuary	50.2451	−5.0249	2005	2005	1	1	1	0	1	1	0	1	1	1	1	1	1	1	1
Fal Estuary	50.2569	−5.0120	2005	2005	1	1	1	0	1	1	0	1	1	1	1	1	1	1	1
Fal Estuary	50.2292	−5.0153	2006	2006	1	1	1	0	1	1	0	1	1	1	1	1	1	1	1
Fal Estuary	50.2270	−4.9849	2005	2005	1	1	1	0	1	1	0	1	1	1	1	1	1	1	1
Fal Estuary	50.1954	−5.0417	2006	2006	1	1	1	0	1	1	0	1	1	1	1	1	1	1	1
North Sea	51.3866	0.9808	2012	2012	1	1	0	1	1	0	1	1	1	1	1	1	1	1	1
Helford River Estuary	50.0936	−5.1332	2005	2005	1	1	1	0	1	1	0	1	1	1	1	1	1	1	1
Fal Estuary	50.1830	−5.0157	2006	2006	1	1	1	0	1	1	0	1	1	1	1	1	1	1	1
Channel	50.7551	0.0404	2011	2011	1	1	0	1	1	0	1	1	1	1	1	1	1	1	1
Fal Estuary	50.1578	−5.0130	2006	2006	1	1	1	0	1	1	0	1	1	1	1	1	1	1	1
Fal Estuary	50.1613	−5.0053	2006	2006	1	1	1	0	1	1	0	1	1	1	1	1	1	1	1
Fal Estuary	50.1735	−4.9953	2006	2006	1	1	1	0	1	1	0	1	1	1	1	1	1	1	1
Fal Estuary	50.1675	−5.0010	2006	2006	1	1	1	0	1	1	0	1	1	1	1	1	1	1	1
Fal Estuary	50.1665	−4.9951	2006	2006	1	1	1	0	1	1	0	1	1	1	1	1	1	1	1
Fal Estuary	50.1616	−4.9920	2006	2006	1	1	1	0	1	1	0	1	1	1	1	1	1	1	1
Fal Estuary	50.1519	−5.0040	2006	2006	1	1	1	0	1	1	0	1	1	1	1	1	1	1	1
Helford River Estuary	50.0864	−5.1877	2005	2005	1	1	1	0	1	1	0	1	1	1	1	1	1	1	1
Channel	50.3613	−4.3479	2010	2010	1	1	1	0	1	1	0	1	1	1	1	1	1	1	1
Plymouth	50.3390	−4.1514	2011	2011	1	1	0	1	1	0	1	1	1	1	1	1	1	1	1
Helford River Estuary	50.0934	−5.2062	2005	2005	1	1	1	0	1	1	0	1	1	1	1	1	1	1	1
Helford River Estuary	50.1017	−5.1645	2005	2005	1	1	1	0	1	1	0	1	1	1	1	1	1	1	1
Dart-Exe-Axe River Estuaries	50.3930	−3.5911	2011	2011	1	1	0	1	1	0	1	1	1	1	1	1	1	1	1
Helford River Estuary	50.1057	−5.1413	2005	2005	1	1	1	0	1	1	0	1	1	1	1	1	1	1	1
Dart-Exe-Axe River Estuaries	50.6664	−3.4642	2013	2013	1	1	0	1	1	0	1	1	1	1	1	1	1	1	1
Channel	50.7313	−2.9011	1997	1997	1	0	1	0	1	1	0	1	1	1	1	0	1	1	1
Channel	50.6185	−2.5385	2004	2004	1	1	1	0	1	0	0	1	1	1	1	0	1	1	1
Channel	50.5739	−2.4533	1997	1997	1	0	1	0	1	1	0	1	1	1	1	0	1	1	1
Channel	50.6152	−1.9547	1997	1997	1	0	1	0	1	1	0	1	1	1	1	0	1	1	1
Poole Harbour	50.6656	−1.9702	2005	2005	1	1	1	0	1	0	0	1	1	1	1	0	1	1	1
Poole Harbour	50.6728	−1.9943	2004	2004	1	1	1	0	1	0	0	1	1	1	1	0	1	1	1
Poole Harbour	50.6782	−2.0028	2004	2004	1	1	1	0	1	0	0	1	1	1	1	0	1	1	1
Poole Harbour	50.6907	−2.0651	2004	2004	1	1	1	0	1	0	0	1	1	1	1	0	1	1	1
Poole Harbour	50.6871	−2.0806	2004	2004	1	1	1	0	1	0	0	1	1	1	1	0	1	1	1
Poole Harbour	50.7006	−2.0651	2004	2004	1	1	1	0	1	0	0	1	1	1	1	0	1	1	1
Poole Harbour	50.7141	−2.0580	2004	2004	1	1	1	0	1	0	0	1	1	1	1	0	1	1	1
Poole Harbour	50.7276	−2.0439	2005	2005	1	1	1	0	1	0	0	1	1	1	1	0	1	1	1
Poole Harbour	50.7231	−2.0368	2004	2004	1	1	1	0	1	0	0	1	1	1	1	0	1	1	1
Poole Harbour	50.7006	−2.0014	1997	1997	1	0	1	0	1	1	0	1	1	1	1	0	1	1	1
Poole Harbour	50.7195	−2.0014	2004	2004	1	1	1	0	1	0	0	1	1	1	1	0	1	1	1
Poole Harbour	50.7276	−2.0042	2004	2004	1	1	1	0	1	0	0	1	1	1	1	0	1	1	1
Poole Harbour	50.7348	−1.9971	2004	2004	1	1	1	0	1	0	0	1	1	1	1	0	1	1	1
Poole Harbour	50.7195	−1.9858	2005	2005	1	1	1	0	1	0	0	1	1	1	1	0	1	1	1
Solent	50.8909	−1.3853	1996	1996	0	1	1	0	1	1	0	1	1	1	1	0	1	1	1
Solent	50.7095	−1.5122	1996	1996	0	1	1	0	1	1	0	1	1	1	1	0	1	1	1
Solent	50.7644	−1.3279	1996	1996	0	1	1	0	1	1	0	1	1	1	1	0	1	1	1
Solent	50.7327	−1.1625	1996	1996	0	1	1	0	1	1	0	1	1	1	1	0	1	1	1
Solent	50.6888	−1.0699	1996	1996	0	1	1	0	1	1	0	1	1	1	1	0	1	1	1
Solent	50.6606	−1.1355	1996	1996	0	1	1	0	1	1	0	1	1	1	1	0	1	1	1
Channel	50.7742	−0.3349	1996	1996	0	1	1	0	1	1	0	1	1	1	1	0	1	1	1
Channel	50.9496	0.7292	2006	2006	1	1	1	0	1	0	0	1	1	1	1	0	1	1	1
North Sea	51.3877	1.4961	1996	1996	0	1	1	0	1	1	0	1	1	1	1	0	1	1	1
North Sea	51.3938	1.4255	1996	1996	0	1	1	0	1	1	0	1	1	1	1	0	1	1	1
Channel	50.0641	−5.2878	1998	1998	1	1	1	0	1	1	0	0	1	1	1	0	1	1	1
Fal Estuary	50.1872	−5.0412	2006	2006	1	1	1	0	1	0	0	1	1	1	1	0	1	1	1
Plymouth	50.2956	−4.1633	1994	1994	1	1	1	0	1	0	0	1	1	1	1	0	1	1	1
Solent	50.9085	−1.3813	1995	1995	1	1	1	0	1	0	0	0	1	1	1	0	1	1	1
Fal Estuary	50.1629	−5.0397	1995	1995	1	1	0	0	1	1	0	0	1	1	1	0	1	1	1
Plymouth	50.4649	−4.1501	1989	1989	0	1	1	0	1	1	0	0	1	1	1	0	1	1	1
Dart-Exe-Axe River Estuaries	50.7147	−3.0580	1989	1989	0	1	1	0	1	0	0	0	1	1	1	0	1	1	1
Solent	50.8482	−1.3650	1994	1994	0	1	1	0	1	0	0	0	1	1	1	0	1	1	1
Bideford Bay Estuary	51.0933	−4.1674	1989	1989	0	1	1	0	1	0	0	0	1	1	1	0	1	1	1
Bideford Bay Estuary	51.1001	−4.1647	1989	1989	0	1	1	0	1	0	0	0	1	1	1	0	1	1	1
Bideford Bay Estuary	51.0924	−4.1205	1989	1989	0	1	1	0	1	0	0	0	1	1	1	0	1	1	1
Bideford Bay Estuary	51.0822	−4.0858	1989	1989	0	1	1	0	1	0	0	0	1	1	1	0	1	1	1
Bideford Bay Estuary	51.0816	−4.0686	1994	1994	0	1	0	0	1	0	0	1	1	1	1	0	1	1	1
Bideford Bay Estuary	51.0180	−4.2027	1989	1989	0	1	1	0	1	0	0	0	1	1	1	0	1	1	1
Bideford Bay Estuary	51.0243	−4.2015	1989	1989	0	1	1	0	1	0	0	0	1	1	1	0	1	1	1
Fal Estuary	50.2067	−5.0355	1992	1992	1	0	0	0	1	1	0	0	1	1	1	0	1	1	1
Fal Estuary	50.1888	−5.0378	1992	1992	1	0	0	0	1	1	0	0	1	1	1	0	1	1	1
Channel	50.3272	−4.6384	2002	2002	1	1	0	0	1	1	0	0	1	0	1	0	1	1	1
Channel	50.4637	−3.4793	1991	1991	0	1	1	0	1	0	0	0	1	1	1	0	1	1	1
Dart-Exe-Axe River Estuaries	50.6899	−3.4785	1989	1989	0	1	1	0	1	0	0	0	1	1	1	0	1	1	1
Medway Estuary	51.3144	0.4583	1997	2010	0	14	0	0	0	0	0	14	0	0	0	0	14	3	0
Celtic Sea	50.4261	−5.0955	2010	2011	0	2	0	0	0	0	0	2	0	0	0	0	2	0	0
Plymouth	50.3129	−4.0519	2001	2011	0	3	0	0	0	0	0	0	0	0	0	0	3	0	0
Channel	50.2237	−3.7804	2009	2011	0	0	0	0	0	0	0	3	0	0	0	0	3	0	0
Plymouth	50.3111	−4.0532	2010	2011	0	2	0	0	0	0	0	0	0	0	0	0	2	0	0
Bristol Channel	51.4810	−2.6782	2003	2003	0	1	0	0	0	0	0	0	0	0	0	0	1	0	0
Plymouth	50.3118	−4.0531	1998	1998	0	0	0	0	1	0	0	0	0	0	0	0	1	0	0
B. number of monitoring years in open sea sites, ordered according to the median, then the mean, then the maximum of monitored years. Only the total digestion technique (td) was applied for open sea sites.																			

Channel	50.4300	−3.1217	1999	2012	13	12	0	12	13	0	12	12	13	13	14	12	14	13	13
Celtic Sea	51.2499	−5.9995	1999	2012	11	11	0	11	11	0	10	11	11	11	12	10	12	11	11
Celtic Sea	48.7990	−8.2450	2009	2009	1	1	0	1	1	0	1	1	1	1	1	1	1	1	1
Celtic Sea	49.9400	−7.1680	2009	2009	1	1	0	1	1	0	1	1	1	1	1	1	1	1	1
Channel	49.9470	−5.5580	2009	2009	1	1	0	1	1	0	1	1	1	1	1	1	1	1	1
Celtic Sea	50.0000	−8.0000	2012	2012	1	1	0	1	1	0	1	1	1	1	1	1	1	1	1
Celtic Sea	50.0000	−6.0000	2012	2012	1	1	0	1	1	0	1	1	1	1	1	1	1	1	1
Celtic Sea	50.0270	−7.8780	2009	2009	1	1	0	1	1	0	1	1	1	1	1	1	1	1	1
Channel	50.2290	−4.5460	2009	2009	1	1	0	1	1	0	1	1	1	1	1	1	1	1	1
Channel	50.3070	−3.2860	2009	2009	1	1	0	1	1	0	1	1	1	1	1	1	1	1	1
Channel	50.3180	−3.0750	2009	2009	1	1	0	1	1	0	1	1	1	1	1	1	1	1	1
Celtic Sea	48.8220	−7.8580	2009	2009	1	1	0	1	1	0	1	1	1	1	1	1	1	1	1
Celtic Sea	50.3630	−6.4860	2008	2008	1	1	0	1	1	0	1	1	1	1	1	1	1	1	1
Channel	50.3710	−3.3980	2009	2009	1	1	0	1	1	0	1	1	1	1	1	1	1	1	1
Celtic Sea	50.3920	−6.9870	2008	2008	1	1	0	1	1	0	1	1	1	1	1	1	1	1	1
Channel	50.3990	−3.2780	2009	2009	1	1	0	1	1	0	1	1	1	1	1	1	1	1	1
Celtic Sea	50.4070	−5.9030	2008	2008	1	1	0	1	1	0	1	1	1	1	1	1	1	1	1
Celtic Sea	50.4490	−7.1720	2008	2008	1	1	0	1	1	0	1	1	1	1	1	1	1	1	1
Celtic Sea	49.2530	−6.2300	2009	2009	1	1	0	1	1	0	1	1	1	1	1	1	1	1	1
Celtic Sea	50.4630	−7.0590	2008	2008	1	1	0	1	1	0	1	1	1	1	1	1	1	1	1
Celtic Sea	50.4750	−7.2970	2008	2008	1	1	0	1	1	0	1	1	1	1	1	1	1	1	1
Celtic Sea	50.5000	−6.0000	2012	2012	1	1	0	1	1	0	1	1	1	1	1	1	1	1	1
Channel	50.5270	−3.2200	2011	2011	1	1	0	1	1	0	1	1	1	1	1	1	1	1	1
Channel	50.5510	−0.1330	2009	2009	1	1	0	1	1	0	1	1	1	1	1	1	1	1	1
Channel	50.5530	−3.0900	2009	2009	1	1	0	1	1	0	1	1	1	1	1	1	1	1	1
Celtic Sea	50.5550	−7.1700	2008	2008	1	1	0	1	1	0	1	1	1	1	1	1	1	1	1
Celtic Sea	50.5600	−7.3070	2008	2008	1	1	0	1	1	0	1	1	1	1	1	1	1	1	1
Channel	50.5640	−2.3150	2009	2009	1	1	0	1	1	0	1	1	1	1	1	1	1	1	1
Celtic Sea	49.2600	−5.3650	2009	2009	1	1	0	1	1	0	1	1	1	1	1	1	1	1	1
Celtic Sea	50.5860	−6.9840	2008	2008	1	1	0	1	1	0	1	1	1	1	1	1	1	1	1
Celtic Sea	50.5870	−6.3950	2008	2008	1	1	0	1	1	0	1	1	1	1	1	1	1	1	1
Celtic Sea	50.6010	−6.9030	2008	2008	1	1	0	1	1	0	1	1	1	1	1	1	1	1	1
Celtic Sea	50.6040	−7.3280	2008	2008	1	1	0	1	1	0	1	1	1	1	1	1	1	1	1
Celtic Sea	50.6200	−7.3230	2008	2008	1	1	0	1	1	0	1	1	1	1	1	1	1	1	1
Channel	50.6500	−0.6160	2009	2009	1	1	0	1	1	0	1	1	1	1	1	1	1	1	1
Celtic Sea	50.6770	−4.8630	2008	2008	1	1	0	1	1	0	1	1	1	1	1	1	1	1	1
Celtic Sea	49.2950	−7.0930	2009	2009	1	1	0	1	1	0	1	1	1	1	1	1	1	1	1
Celtic Sea	50.7500	−5.5000	2012	2012	1	1	0	1	1	0	1	1	1	1	1	1	1	1	1
Celtic Sea	50.9080	−5.9410	2008	2008	1	1	0	1	1	0	1	1	1	1	1	1	1	1	1
Celtic Sea	49.7680	−8.5220	2009	2009	1	1	0	1	1	0	1	1	1	1	1	1	1	1	1
Celtic Sea	50.9830	−6.2500	2008	2008	1	1	0	1	1	0	1	1	1	1	1	1	1	1	1
Celtic Sea	50.9830	−6.1350	2008	2008	1	1	0	1	1	0	1	1	1	1	1	1	1	1	1
Celtic Sea	51.0000	−6.0000	2012	2012	1	1	0	1	1	0	1	1	1	1	1	1	1	1	1
Celtic Sea	51.0000	−5.0000	2012	2012	1	1	0	1	1	0	1	1	1	1	1	1	1	1	1
Celtic Sea	51.0180	−6.3270	2008	2008	1	1	0	1	1	0	1	1	1	1	1	1	1	1	1
Bristol Channel	51.1090	−4.3560	2008	2008	1	1	0	1	1	0	1	1	1	1	1	1	1	1	1
Celtic Sea	51.1160	−6.1430	2008	2008	1	1	0	1	1	0	1	1	1	1	1	1	1	1	1
Channel	49.8080	−4.1960	2009	2009	1	1	0	1	1	0	1	1	1	1	1	1	1	1	1
Celtic Sea	51.1440	−6.7470	2008	2008	1	1	0	1	1	0	1	1	1	1	1	1	1	1	1
Celtic Sea	51.1870	−6.1490	2008	2008	1	1	0	1	1	0	1	1	1	1	1	1	1	1	1
Celtic Sea	51.2080	−6.2530	2008	2008	1	1	0	1	1	0	1	1	1	1	1	1	1	1	1
Celtic Sea	51.2110	−6.1920	2008	2008	1	1	0	1	1	0	1	1	1	1	1	1	1	1	1
Celtic Sea	51.2500	−6.5000	2012	2012	1	1	0	1	1	0	1	1	1	1	1	1	1	1	1
Celtic Sea	51.2500	−5.5000	2012	2012	1	1	0	1	1	0	1	1	1	1	1	1	1	1	1
Celtic Sea	51.3290	−6.2980	2008	2008	1	1	0	1	1	0	1	1	1	1	1	1	1	1	1
Channel	49.8440	−3.8330	2009	2009	1	1	0	1	1	0	1	1	1	1	1	1	1	1	1
Celtic Sea	51.4250	−5.9400	2008	2008	1	1	0	1	1	0	1	1	1	1	1	1	1	1	1
Celtic Sea	51.4550	−5.8380	2008	2008	1	1	0	1	1	0	1	1	1	1	1	1	1	1	1
Celtic Sea	51.4810	−6.1950	2008	2008	1	1	0	1	1	0	1	1	1	1	1	1	1	1	1
Celtic Sea	51.4890	−6.0340	2008	2008	1	1	0	1	1	0	1	1	1	1	1	1	1	1	1
Celtic Sea	51.5000	−6.0000	2012	2012	1	1	0	1	1	0	1	1	1	1	1	1	1	1	1
Bristol Channel	51.5070	−4.6690	2008	2008	1	1	0	1	1	0	1	1	1	1	1	1	1	1	1
Celtic Sea	49.8530	−6.0250	2009	2009	1	1	0	1	1	0	1	1	1	1	1	1	1	1	1
Celtic Sea	51.5540	−5.9500	2008	2008	1	1	0	1	1	0	1	1	1	1	1	1	1	1	1
North Sea	51.5732	1.9716	2011	2011	1	1	0	1	1	0	1	1	1	1	1	1	1	1	1
Celtic Sea	51.5760	−6.1420	2008	2008	1	1	0	1	1	0	1	1	1	1	1	1	1	1	1
Channel	50.6660	−1.6780	2009	2009	1	1	0	1	1	0	0	1	1	1	1	0	1	1	1
Channel	50.8860	1.0800	2009	2009	1	1	0	1	1	0	0	1	1	1	1	0	1	1	1

C. Number of sites monitored for six years or more.																			

nb of years	As	Cd	Cr (sad)	Cr (td)	Cu	Fe (sad)	Fe (td)	Hg	Ni	Pb	Zn								

>= 6	49	70	50	9	59	44	9	67	59	59	59								
>= 7	47	63	45	8	53	42	8	62	53	52	53								
>= 8	40	59	42	7	50	41	7	59	49	49	50								
>= 9	29	52	35	6	46	34	6	54	45	45	46								
>= 10	17	49	32	5	42	29	5	51	42	42	42

**Table 2:** Number of sites monitored yearly for trace element contamination in very fine sediments. Digestion techniques: strong acid digestion (sad) or total digestion (td) are specified and considered separately for Cr and Fe.

YEAR	As	Cd	Cr (sad)	Cr (td)	Cu	Fe (sad)	Fe (td)	Hg	Ni	Pb	Zn
A. Coastal sites, from 1983 to 2013 (no data for years 1985 and 1986).											

1983	4	4	4	0	4	0	0	4	4	4	4
1984	2	2	2	0	2	0	0	2	2	2	2
1987	0	2	2	0	2	1	0	0	2	2	2
1988	0	0	2	0	2	0	0	1	2	2	2
1989	1	15	14	0	14	2	0	0	14	14	14
1990	2	7	5	0	7	1	0	2	7	7	7
1991	6	27	27	0	27	2	0	4	27	26	26
1992	16	14	13	0	23	18	0	10	23	23	23
1993	29	50	50	0	49	23	0	29	50	50	50
1994	23	25	24	0	25	2	0	25	25	25	25
1995	24	44	36	0	45	27	0	37	44	44	44
1996	21	60	59	0	60	42	0	61	60	60	60
1997	10	42	46	0	46	44	0	50	46	46	46
1998	5	43	40	0	41	39	0	45	40	40	40
1999	27	51	43	6	50	43	6	52	49	49	49
2000	16	55	41	6	47	40	6	53	47	47	47
2001	39	59	45	6	51	44	6	55	51	51	51
2002	50	56	43	7	51	43	7	54	51	50	50
2003	48	56	43	6	49	41	7	54	49	49	49
2004	68	77	62	7	69	45	6	75	68	69	69
2005	72	74	66	7	72	57	7	76	73	73	73
2006	76	80	71	5	77	69	5	80	76	76	76
2007	49	52	38	10	49	37	10	56	48	48	48
2008	49	61	38	12	50	35	12	61	50	50	50
2009	13	43	7	6	13	6	6	42	12	13	13
2010	18	53	12	6	18	7	6	47	18	18	18
2011	21	52	5	15	20	6	15	45	21	19	19
2012	9	9	2	7	9	0	7	9	9	9	9
2013	8	8	2	6	8	2	6	8	8	8	8

B. Open sea sites, from 1999 to 2012.											

1999	2	2	0	2	2	0	0	2	2	2	2
2000	1	0	0	0	1	0	1	0	1	1	1
2001	2	2	0	2	2	0	2	2	2	2	2
2002	2	2	0	2	2	0	2	2	2	2	2
2003	0	0	0	0	0	0	2	0	0	0	0
2004	2	2	0	2	2	0	0	2	2	2	2
2005	2	2	0	2	2	0	2	2	2	2	2
2006	2	2	0	2	2	0	2	2	2	2	2
2007	2	2	0	2	2	0	2	2	2	2	2
2008	34	34	0	34	34	0	34	34	34	34	34
2009	25	25	0	25	25	0	23	25	25	25	25
2010	2	2	0	2	2	0	2	2	2	2	2
2011	3	3	0	3	3	0	3	3	3	3	3
2012	11	11	0	11	11	0	11	11	11	11	11

The overall aim of this paper is to give ecotoxicologists, coastal ecologists (practitioners, scientists and policy makers) and government decision makers a ready-to-use .csv database detailing elemental composition of sediments (http://dx.doi.org/10.17632/m68k63nnk3.1), useful for local, regional and global case studies. The data for nine trace elements (TEs; As, Cd, Cr, Cu, Fe, Hg, Ni, Pb and Zn), represent 87% of the database and all (except Fe) are included on the US EPA priority pollutant list [3]. The nine TEs have on average 4434 ± 607 SD sedimentary - silt and clay [4] - measurements from 320 of the 334 sites. In addition, data detailing 20 supplementary elements (Mn, Al, Li, Sn [including tributyltin (TBT)], Ba, Sb, B, Ca, Mo, Co, Se, K, Mg, Be, V, Ti, Na, Ag, Tl and Sr) are included. To provide end-users with the relevant context on spatial and temporal coverage, monitoring statistics are given for the nine TEs in Table 1. These include the first and last year of sediment monitoring, the number of years monitored, and summary statistics showing minimum, maximum, mean and median numbers of years monitored. Statistics are given separately for coastal (Table 1A) and open sea (Table 1B) sites. Two columns for Fe and Cr are included because the extraction effiency differs according to the technique used [total hydrofluoric (td) or strong acid (sad) digestion, see Experimental Design, Materials and Methods section for details]. Statistics on the number of sites monitored for six years and more are given for each of the nine TEs (Table 1C). Table 1 also contains site geographic coordinates and their regional location (Area). Table 1, used in conjunction with the map of the sites (Supplementary Materials), allows for site identification and elements for which the monitoring effort was greatest, without having to analyse the complete .csv database. Finally, Table 2 gives, for each of the nine TEs (td and sad techniques considered separately for Cr and Fe), the number of sites monitored per year, from the first 1983 records to 2013. Statistics are given separately for coastal (Table 2A) and open sea (Table 2B) sites. There were no Southern England coastal sites monitored for sediment elemental concentrations for years 1985 and 1986 (and globally few sites for years 1983 to 1990; Table 2A); and open sea sites were monitored mainly in the years 2008, 2009 and 2012 (Table 2B).

## 2 Experimental Design, Materials and Methods

### 2.1 Mining public databases

#### 2.1.1 Database selection and data processing

UK coastal monitoring data on elemental concentrations in sediment samples are held in two key public repositories: a) the EA and b) the MERMAN database managed by the BODC under the Clean Safe Seas Environmental Monitoring Programme (CSEMP) [5]. From these a merged EA-MERMAN database focused on the Channel, southern North and Celtic Seas was generated [using Microsoft Excel (Microsoft Corporation, US), R [6] in RStudio [7] and Google Earth (Google LLC)]. Based on detailed lists of the ‘Determinands’ sent by the BODC (e.g., ‘chromium’, ‘mercury’) and ‘Determinands’ (e.g. ‘Arsenic, <63 µm: Dry Wt’, ‘Cadmium: HF Digest: Dry Wt’) and ‘Sample Point Types’ (e.g. ‘saline water - estuarine sites - non bathing/shellfish’, ‘miscellaneous environment – sediments’) sent by the EA, elementary concentration data: i) for all UK marine waters from the BODC; and ii) from the two regions South England and South-West England from the EA were requested.

Data stored in the MERMAN database are dedicated to marine waters only (coastal and open sea waters) from 1999 onwards. They were provided as a list of files (.xls(x) extension files), each corresponding to one year of sediment monitoring with a unique ‘Determinand Full Name’ per element. Georeferenced MERMAN sites (WGS84 coordinates) were projected on to maps (Google Earth projection, Google LLC) and subsampled northwards from the Celtic Sea to the Bristol Channel and Thames Estuary. To one site there corresponded several georeferenced sampling points, resulting in multiple close coordinates for the same location. These multiple coordinates were averaged so that each site only corresponded to a unique geolocation. The resulting secondary MERMAN dataset for Southern England contained sediment concentration data of thirteen TEs: Al, As, Cd, Cr, Cu, Fe, Pb, Li, Mn, Hg, Ni, V and Zn from 95 sites, for a total of 12,540 data points.

The two South and South West England datasets sent by the EA (codes SO and SW, held in one dataset separated by region [.mdb extension files]) contained elemental concentration data from the mid-1980s. To avoid losing any data along the transition from estuarine to coastal water continuum, both inland and marine site data were requested. Georeferenced EA sites were projected on to maps (Google Earth), after coordinate transformation from OSGB36 format to WGS84 for ease of use with Google Earth and ArcGis (Esri, Redlands, CA). Only sites in transitional, estuarine and coastal waters were considered; i.e. all sites under marine and tidal influence (terrestrial and freshwater sites were discarded). We considered that geographic coordinates of EA-sampled sites were correct, since we selected sites according to their geographic position; although in earlier sampling years, site coordinates corresponded sometimes/often to the highest point of the tide on the shoreline. Nowadays, EA site coordinates correspond to sampling locations within water bodies (EA, C. Ashcroft pers. com.).

Early EA records preceded computer-recording processes, which sometimes resulted in a mismatch of sample material codes with more recent records. We, therefore, selected EA data as follows. Each data, for previously georeferenced selected sites only, had a unique material description identifier, or ‘MATERIAL_DESC’. EA data with a ‘MATERIAL_DESC’ related to sediments: ‘ESTUARY SEDIMENT’, ‘ESTUARY SEDIMENT - <63UM FRACTION’, ‘COASTAL / MARINE SEDIMENT’, ‘RUNNING SURFACE WATER SEDIMENT - <63UM FRACTION’, ‘RUNNING SURFACE WATER SEDIMENT’, ‘ESTUARY SEDIMENT - INTER TIDAL’, ‘ESTUARY SEDIMENT - SUB TIDAL’, ‘COASTAL / MARINE SEDIMENT - <63UM FRACTION’ and ‘ESTUARY SEDIMENT - INTER TIDAL - <63UM FRACTION’ were first selected. Limiting our request to these nine sediment identifiers, would have missed the older data for which the ‘MATERIAL_DESC’ identifier hadn't been properly encoded. Because the main objective of the co-submitted Environment International paper [1] was to investigate the temporal trend of TE contamination, additional data with material identifiers not linked to sediments were also selected. From an extended dialogue and question and answer process with the EA, we were able to select additional sediment elemental concentration data, belonging to nine supplementary ‘MATERIAL_DESC’ such as ‘UNCODED’, ‘SOIL’, ‘SEA WATER’ etc. This time consuming, but necessary, approach required checks of all selected mapped sites for concentration data that did not correspond directly to one of the sediment-related identifiers. If any doubt remained for sites and/or an elemental concentration data points, they were discarded from the final filtered EA dataset. The data filtering related to sediment identifiers only, resulted in a dataset of 30,395 data; but with the supplementary nine MATERIAL_DESC identifiers, we generated a dataset of 39,910 data, i.e*.* 31% larger, for twenty-nine chemicals (including the thirteen in the MERMAN dataset), in 254 sites. These chemicals were: As, Cd, Cr, Cu, Pb, Hg, Ni, Zn, Fe, Mn, Al, Li, Sn [including tributyltin (TBT)], Ba, Sb, B, Ca, Mo, Co, Se, K, Mg, Be, V, Ti, Na, Ag, Tl and Sr.

#### 2.1.2 Generating a new database on sediment elemental concentrations for Southern England

The resulting EA and MERMAN datasets were merged into a unique EA-MERMAN database, after harmonization of the names of shared variables (e.g. latitude variable was labelled ‘X‘ in the EA dataset, ‘Sample.Latitude‘ in the MERMAN dataset). The EA dataset contained 21 variables, the MERMAN dataset 23 variables, the merged database 29 variables, subsequently in this were included three new variables created for data analysis. In particular, a new code was assigned to each site with an identifier for EA or MERMAN origin [‘SITEnb_db’ variable with a unique number/letter (‘EA‘ or ‘ME‘)]. Elemental concentration units were standardized. Some concentrations were in ppm, others in ppb or in %, with differences of units between datasets. Some coastal sites data were also duplicated between the two EA and MERMAN datasets; these were removed, giving priority to the EA data (the time series dataset).

Once merged, the resulting EA-MERMAN database consisted of 45,962 data-points, from 334 sites, for twenty-nine chemicals over 31 years (1983–2013) of environmental monitoring. The twenty-nine chemical full database is saved in a ready-to-use .csv format for further analysis (http://dx.doi.org/10.17632/m68k63nnk3.1). For the co-submitted Environment International paper [1] we selected the nine most monitored TEs, namely Cu, Zn, As, Cd, Cr, Fe, Hg, Ni and Pb together representing 87% of data (320 sites). Subsequent data analysis for this subset is fully detailed in [1].

### 2.2 Background detail on sample processing and trace element analysis

All competent UK authorities undertaking monitoring (should) use the same programme monitoring manual, the ‘Clean Seas Environment Monitoring Programme (CSEMP) - Green Book’ [5]. Whilst, detailed information on procedural guidelines for sediment TE analysis is available in the Appendices 6 and 7 of the Green Book, we have provided relevant summary information here for context during the EA and MERMAN database usage. The shared analytical considerations of the secondary, compiled EA-MERMAN database are the concentration unit (mg kg_dw_^−1^) and, for the nine TEs the median detection limit values (DL, in mg kg_dw_^−1^): 0.1 for Cd, Cr (sad) and Hg, 1 for Cu, 2 for As, 3 for Fe (sad), 5 for Ni and Zn and 8 for Pb. For TE concentrations below the analytical procedure DL, we used half the DL values [2].

#### 2.2.1 MERMAN samples

Sediment TE concentration data stored in the MERMAN database are acquired following the Green Book guidelines. Briefly, sediment samples are wet or freeze-dry sieved through a nylon 63 µm mesh, and the <63 µm silt and clay fraction [4], i.e. the grain size fraction that accumulates contaminants is retained for analysis (a small minority of MERMAN sediment samples were ‘untreated’, thus removed from the analysis). A total digestion procedure, most often hydrofluoric acid (HF) digestion, is required to allow data to be normalized (e.g. to Al or Li) to facilitate inter-site comparison of anthropogenic contamination levels. A partial extraction method is acceptable for determination of long-term trends at sites where this method has traditionally been used (see the case of the EA database below). The analytical technique chosen is not mandatory, but most laboratories now use Inductively Coupled Plasma Mass Spectrometry (ICP-MS) for TE determination. Hg can be determined by cold vapour atomic absorption spectrometry or atomic fluorescence.

#### 2.2.2 EA samples

For EA samples, the protocol has evolved since the acquisition of the oldest (1980s) data. EA sediment samples were formerly wet sieved and the <90 µm fraction retained, but this was changed to the <63 µm fraction in the 1990s; a small minority of samples were sieved through a 2000 µm mesh size, thus were removed from the analysis. Sediment requiring analysis for TE contaminants would have been analysed following digestion with hot nitric acid (HNO_3_) or aqua regia (sad technique). The intention would have been to maximise the extractable TE components that could be considered as bioavailable [8] in the environment. A partial extraction method is - according to the Green Book - acceptable for determination of long term trends at sites where this method has traditionally been used (see above), which was the aim of the present analysis and co-submitted [1] paper that studies TE concentration trends over time. For more recent sediment samples acquired in the framework of the CSEMP programme (see above), stored in a duplicate way between the EA and BODC databases, the digestion technique, using HF acid, is total. These data are distinguishable in the EA database by using unique ‘Determinand’ codes with reference to HF (e.g. ‘Lead: HF Digest: Dry Wt’). HF, unlike hot HNO_3_/aqua regia, solubilizes TEs bound to silicate structures. We have considered hot HNO_3_/aqua regia and HF digestions equivalent for Cu, Zn, As, Cd, Hg, Ni and Pb since those TEs are a small part of the sediment matrix; confirmed by comparing extraction efficiencies [9;10]. For Fe and Cr, differences in sediment extraction efficiencies were >10%, therefore, data from each extraction procedure were analysed separately. Finally, quality assurance processes also ensured there was no step-change effect for changeover of analytical technique, e.g. flame atomic absorption to ICP-MS (most laboratories); Hg would be analysed with cold vapour technique (EA, C. Ashcroft pers. comm.).

## Ethics Statement

Out of scope.

## CRediT Author Statement

**Richir Jonathan:** Conceptualization, Data curation, Formal analysis, Investigation, Methodology, Supervision, Validation, Visualization, Writing - original draft, Writing - review & editing; **Bray Simon:** Methodology, Validation, Writing - review & editing; **McAleese Tom:** Investigation; **Watson Gordon J.:** Conceptualization, Funding acquisition, Investigation, Methodology, Project administration, Resources, Supervision, Validation, Visualization, Writing - original draft, Writing - review & editing.

## Declaration of Competing Interest

The authors declare that they have no known competing financial interests or personal relationships which have or could be perceived to have influenced the work reported in this article.

## Appendix Supplementary materials

Image, application 1

Image, application 2
